# Epidemiological cut-offs for Sensititre susceptibility testing of *Mycobacterium tuberculosis*: interpretive criteria cross validated with whole genome sequencing

**DOI:** 10.1038/s41598-020-57992-x

**Published:** 2020-01-23

**Authors:** Nazir A. Ismail, Farzana Ismail, Lavania Joseph, Netricia Govender, Linsay Blows, Koné Kaniga, Shaheed V. Omar

**Affiliations:** 1 National Institute for Communicable Diseases, Centre for Tuberculosis, Johannesburg, South Africa; 2 Department of Medical Microbiology, University of Pretoria, Pretoria, South Africa; 3 Department of Internal Medicine, University of Witwatersrand, Johannesburg, South Africa; 4 Janssen Research & Development, Titusville, NJ United States of America

**Keywords:** Laboratory techniques and procedures, Molecular medicine

## Abstract

Universal drug susceptibility testing (DST) is an important requirement of the End TB Strategy. The Sensititre broth micro-dilution assay (BMD) tests multiple drugs quantitatively. We defined interpretive criteria for this assay and analysed genotypic-phenotypic relationships. 385 *Mycobacterium tuberculosis* clinical isolates were processed for BMD and whole genome sequencing. The epidemiological cut-off value 99% (ECV^99^) amongst genotypically wild type (gWT) strains defined susceptibility. Minimum inhibitory concentration distributions of the resistance-associated variants (RAVs) for each drug were analysed. Susceptibility (µg/mL) criteria were determined as follows: rifampicin (≤0.125), isoniazid (≤0.25), ethambutol (≤2.0), moxifloxacin (≤0.5), levofloxacin (≤1.0), amikacin (≤2.0), kanamycin (≤8.0), capreomycin (≤4.0), clofazimine (≤0.25) and linezolid (≤2.0). Most drugs showed clear separation between gWT and RAV. Isoniazid showed a tri-modal pattern with 14/17 strains at ECV^99^ harbouring a *fabG1* c. -15C > T RAV. Ethambutol RAVs at *embB* codons 306, 405 and 497 were responsible for resistance and showed differential distributions. Moxifloxacin RAVs (gyrA codon 90) were a dilution or two higher than the ECV^99^ while gyrB RAVs were uncommon and showed drug specific resistance propensity. Interpretive criteria established were robust facilitating progress towards universal DST and individualised precision medicine. This study demonstrates the value of quantitative DST to accurately interpret mutation data.

## Introduction

Tuberculosis (TB) is a major contributor to morbidity and mortality globally and efforts to address this public health threat has shown positive signs of decline in recent years^1^. However, multi-drug resistant TB (MDR-TB) defined as TB with resistance to the core first line drugs, rifampicin and isoniazid, is forecasted to increase into the future^2^. This form of TB is more complex to manage and microbiological testing is required to determine the resistance profile and guide appropriate treatment regimens. Universal drug susceptibility testing (DST) is highlighted as an important component of the World Health Organizations (WHO) End TB strategy^3^. The increased availability of molecular tests that effectively detect resistance associated variants (RAVs) in the 81 bp hotspot of the *rpoB* gene which almost universally confers resistance to rifampicin has been very successful^4^. However, for other drugs, genetic targets and markers are less well defined and testing is not ideal for resistance determination.

Phenotypic drug susceptibility testing (pDST) in contrast provides a wide array of drugs that could be tested, however, such methods are often technically challenging. Testing at a single concentration recommended by the WHO, referred to as a critical concentration (CC)^5^ is the current approach. These CCs were established on less robust criteria and consensus. The use of epidemiological cut-off values (ECVs) are widely used in general microbiology and are preferred. Studies have highlighted the weaknesses of the CC based approach compared to the ECV based approach for *Mycobacterium tuberculosis*(Mtb)^6^ and the ECV criteria are now adopted by WHO^7^.

The limited arsenal of mycobacterial drugs available is driving an emerging need for pDST that could provide levels of resistance allowing continued use of certain drugs in cases of “borderline” or “low-level” resistance with dose adjustment^8^. In addition, drug regimens for TB have historically been established on available knowledge at the time, and lacked pharmacokinetic/pharmacodynamic (PK/PD) assessments that could reliably provide the optimal dosage for effect. Unfortunately, as combination therapy is mandatory for TB, clinical outcome data cannot easily be used to confirm the interpretive criteria.

The Sensititre MYCOTB is a commercial broth microdilution (BMD) assay for *Mycobacterium tuberculosis* MIC testing introduced by TREK Diagnostic Systems (forming part of the Thermo Fischer Scientific group). Multiple drugs are tested at a pre-defined concentration in a 96-well plate. Results are available 14–21 days from inoculation. This method is increasingly being used, and does have the potential to address gaps in achieving universal DST for Mtb. Unfortunately; a robust approach to determine break points for this method has not been established. Results are often interpreted against break points established for other methods including agar proportion and MGIT^9–11^. This is inappropriate and not suitable for clinical use. In addition, no interpretive criteria are provided in the package insert^12^. Furthermore, many of the Mtb ECV studies have not included this method^6,13–15^.

We undertook to determine the ECVs for the customized panel of anti-mycobacterial drugs, and evaluated these against known genotypic resistance determinants as a secondary validation.

## Results

A total of 385 strains were analysable having MIC determined on BMD for all drugs and whole genome sequencing (WGS) data for the genetic targets of interest. The ECVs at 95%, 97.5% and 99.0% were determined for each drug and shown in Table 1, while the graphs used to derive these ECVs are shown in Supplementary Material (Figs. S1–S10). The ECVs at all three values were the same except for isoniazid, kanamycin and clofazimine.

**Table 1:** Epidemiological cut-off values for each drug among fully susceptible strains and interpretive criteria.

Drug	gWT (µg/mL)			
	ECV 95.0%	ECV 97.5%	ECV 99.0%	ECV 99.9%
Rifampicin	0.125	0.125	**0.125**	0.125
Isoniazid	0.125	0.125	**0.25**	0.25
Ethambutol	2	2	**2**	4
Levofloxacin	1	1	**1**	2
Moxifloxacin	0.5	0.5	**0.5**	1
Amikacin	2	2	**2**	4
Kanamycin	4	4	**8**	8
Capreomycin	4	4	**4**	8
Linezolid	2	2	**2**	4
Clofazimine	0.125	0.125	**0.25**	0.5

gWT: genotypically wild type; ECV: epidemiological cut-off value. Bold font: ECV final criteria.

**Figure 1:**
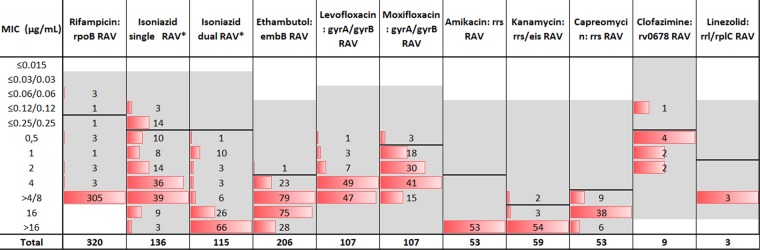
Overall distribution of MICs among RAVs for each drug. MIC: minimum inhibitory concentration. RAV: Resistance associated variant. Shaded area represents the testing range for each drug. The solid line in each distribution is the ECV^99^ that was derived by the ECOFFinder. The gWT distribution plot including raw count and fitted for each drug is shown in Supplementary Figs. 1–10. *Isoniazid RAVs: katG, inhA, fabG1, ahpC, ahpC promoter. Among the injectable drugs, 52 strains had a RAV (NC_000962.3: g.1473246A > G) and 1 strain had a RAV (NC_000962.3: g.1473247C > T). The MIC was >16 for amikacin and kanamycin while for capreomycin the distribution is shown in Fig. 3.

The distribution of MICs among strains with RAVs is shown in Fig. 1. Applying the ECV^99^ to strains with RAVs; the overlap was minimal for most drugs except isoniazid and clofazimine. The misclassification for these two drugs would be 7% and 11% respectively, while for all other drugs misclassification was 3% or lower (Table S1). For clofazimine, the number of resistant strains were low (n = 9) and one of these was misclassified (MIC = 0.12 µg/mL).

For rifampicin the MIC distribution of the RAVs were clearly separated with 95% (305/320) having an MIC > 4 µg/mL. Importantly, the ECV^99^ at 0.125 µg/mL correctly classified the majority of strains including the “disputed” *rpoB* RAVs (99%; 316/320). Drug resistance associated with these RAVs are often missed by the MGIT 960 (Becton Dickinson, USA), a broth based phenotypic method when tested at a CC of 1 µg/mL. Some have suggested that the CC of 1 µg/mL is too high, and have recommended this to be revised^16^.

There were 14/17 misclassified RAVs that had an MIC at the ECV^99^ for isoniazid (0.25 µg/mL) and harboured a RAV in the promoter for the *fabG1-inhA-hemZ* polycistronic operon (*fabG1* promoter) fabG1 c. –15 known to confer low-level resistance (Fig. 1). In contrast all RAVs in *katG* were clearly separated having a modal MIC of 4–8 µg/mL, while those with both a *katG* and *fabG1* promoter RAV had a modal MIC of 16 µg/mL or higher. There were 15 strains with an Ile194Thr RAV in the *inhA* gene and all were clearly in the resistant category, ranging from 0.5 µg/mL to 8 µg/mL.

**Figure 2:**
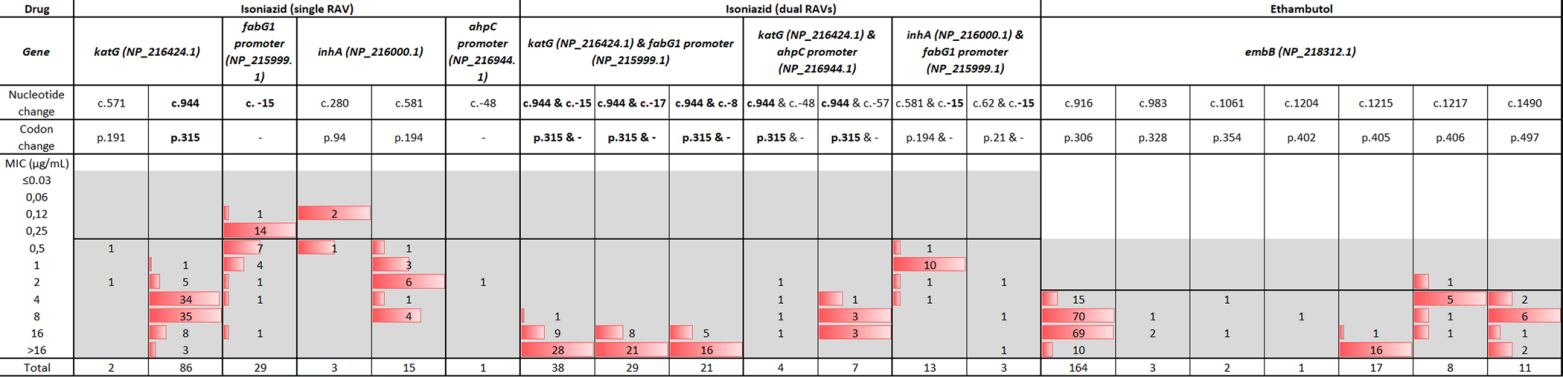
Distribution of MICs and RAVs at specific positions for selected first line drugs. MIC: minimum inhibitory concentration. RAV: Resistance associated variant. Shaded area represents the drug resistant testing range for each drug. The solid line in each distribution is the ECV^99^ that was derived by the ECOFFinder. The gWT distribution plot including raw count and fitted for each drug is shown in Supplementary Figs. 1–10. Nucleotide and codons in bold represent the targets at specific positions included in WHO endorsed Genotype LPA first line assay.

Ethambutol (*embB)* resistance was due to a variety of RAVs in combination. The most dominant RAVs occurred at codons 306, 405 and 497 (Fig. 2), and some of their distributions were close to the ECV^99^ (2 µg/mL) i.e. one two fold dilution above the ECV^99^. These were observed at the following codons: 306 (9%;15/164), 497 (18%;2/11) and 405 (0%;0/17). In contrast, RAVs at codon 406 were commonly observed at MIC of 4 µg/mL (63%; 5/8).

**Figure 3:**
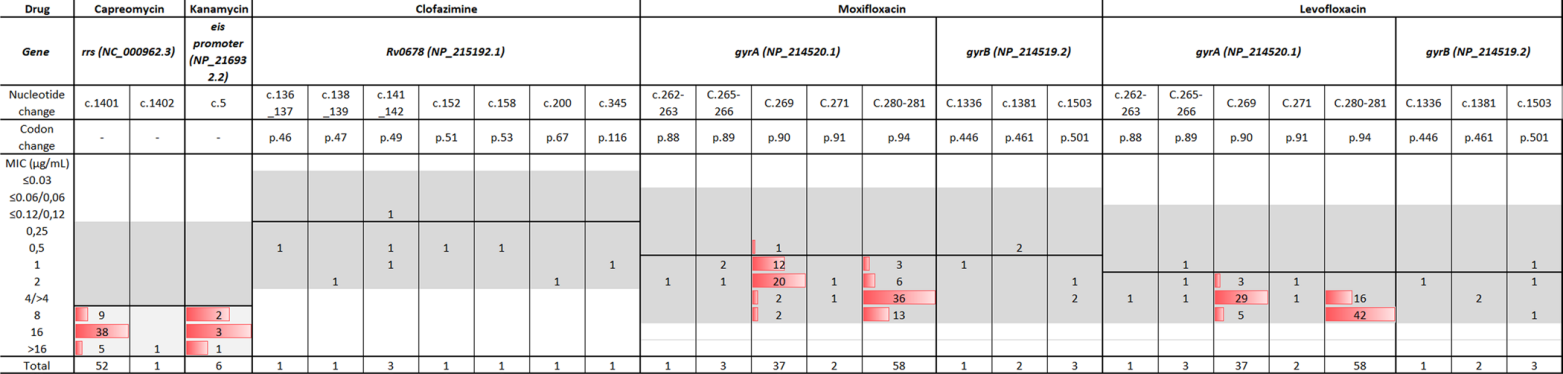
Distribution of MICs and RAVs at specific positions for selected second line drugs. MIC: minimum inhibitory concentration. RAV: Resistance associated variant. Shaded area represents the drug resistant testing range for each drug. The solid line in each distribution is the ECV^99^ that was derived by the ECOFFinder. The gWT distribution plot including raw count and fitted for each drug is shown in Supplementary Figs. 1–10.

Two fluoroquinolones were analysed, levofloxacin and moxifloxacin, both showed minimal misclassification applying the ECV^99^ of 1 µg/mL and 0.5 µg/mL respectively (Fig. 3). The modal MIC for RAVs at *gyrA* codon 94 for levofloxacin was >4 µg/mL, while for moxifloxacin it was 4 µg/mL. For RAVs at other codons the modal MIC was 4 µg/mL and 2 µg/mL respectively. For moxifloxacin, 5% (3/58) of RAVs at *gyrA* codon 94 had an MIC of one twofold dilution above the ECV^99^ with two of the three having a *gyrA* p.Asp94Ala RAV known to confer lower MIC. In contrast, at other hotspot codons, 33% (14/43) of RAVs had MICs of 2 µg/mL, being a dilution above the ECV^99^. When assessing the *gyrB* RAVs that were uncommon, there appeared to be a differential susceptibility pattern between the two drugs. RAVs at codon 501 were consistently resistant to moxifloxacin, while at codon 461 moxifloxacin was consistently susceptible, while levofloxacin was resistant.

Amongst the second line injectable drugs, the *rrs* c. 1401 A > G RAV were common and conferred cross-resistance to all three drugs clearly separating from the gWT distribution (Figs. 1 and 3). For capreomycin, only 10% (5/52) of strains had an MIC > 16 while for the other two drugs it was 100% (52/52). Among RAVs which are known to confer low-level resistance to kanamycin, the *eis promoter* c. −14C > T RAV was identified in six strains, of which 2/6 had an MIC at the ECV^99^.

Linezolid strains with resistance were few (n = 3) and the ECV^99^ was appropriate, correctly classifying the strains (Fig. 1). Similarly, for clofazimine, the number of strains with resistance was also limited. Eight of nine were correctly classified, with one outlier having an MIC of 0.12 µg/mL. It should be noted that the *Rv0678* gene encodes for an efflux pump and has previously been identified to demonstrate higher MICs in those with drug exposure^17^.

## Discussion

General microbiology has for a long time used MIC testing as the standard method for pDST, however, this has been lacking for mycobacteriology. Although the Sensititre MYCOTB assay, a commercial BMD method has been available for many years, a robust study to define interpretative criteria for it has been lacking, limiting its broader utilization. This study has addressed this need applying CLSI standards to derive appropriate interpretive criteria, albeit using a customised panel of drugs. An additional validation was included, comparing the MIC distributions of gWT to known high confidence RAVs. This study, not only formally established criteria for both first and second line drugs for this method, but has also highlighted the important value of quantitative DST for Mtb. The RAV distributions for several gene-drug combinations have MICs close to the ECV^99^ necessitating quantitative DST. Additionally, some specific RAVs conferred either low or high level resistance, raising the potential value for using this method combined with PK/PD measurements to optimise therapies and improve patient outcomes.

A case in point has been ethambutol which is widely used in first line, and now in the WHO endorsed short course MDR-regimen. Reliability of testing this drug has been questioned when applying a single critical concentration^6,13^ and, therefore, pDST is usually not recommended^5^. Empiric use of this drug in the presence of high resistance levels and disabling side effects such as optic neuritis are concerning^18^. Testing for resistance is thus important to ensure patients are benefitted and not harmed. We observed overlapping distributions which explains reproducibility issues when performing DST for this drug. Using the quantitative DST approach resistance and susceptibility are clearly distinguished when not close to the ECV^99^. In the case of RAVs at *embB* codon 306, which were most common and known to confer resistance, 9% had MICs at the ECV^99^. Nonetheless, 91% would be clearly separated aiding appropriate clinical management. It is also interesting to note that strains with RAV at *embB* codon 406 commonly had MICs around the ECV^99^. Thus clinical studies could be targeted addressing this selection of patients to assess the clinical relevance, or alternatively the potential value of a higher dose in these cases, where ethambutol may serve as a useful oral option.

For isoniazid resistance, the distributions appeared tri-modal, split between combined *inhA* & *fabG1 promoter*, *katG* alone and katG & *inhA & fabG1 promoter* combination RAVs. Although molecular testing does provide an indication of low or high level RAVs, it is clear that RAVs with the *fabG1*promoter have a broad MIC distribution ranging between 0.25 µg/mL to 16 µg/mL, and having the actual MIC is valuable. Of interest, was the *fabG1* c. −15C > T RAV, which is detected by WHO endorsed commercial assays, generally having lower MICs with 90% (26/29) having an MIC ≤ 1 µg/mL compared with the *katG* p. Ser315Thr RAV where only 1% (1/86) had an MIC ≤ 1 µg/mL. This has important value in decisions related to the use of high dose isoniazid for treating drug resistant TB as applied in the WHO recommendations^19^. Of note, was the 7% of the *fabG1 promoter* RAVs that fell within the gWT distribution and could potentially be treated with standard dose therapies avoiding dose related peripheral neuropathy. When restricting to the key RAVs included in commercial molecular assays, the sensitivity for molecular detection is 91.6% (230/251), which is consistent with a recent review of RAV data^4,20^. An important RAV that is not included in molecular assays is the *inhA* p. Ile194Thr which accounted for 6% (15/251) of resistance in this study, which, if incorporated could improve molecular test performance significantly (~97.6%).

The fluoroquinolones, levofloxacin and moxifloxacin both had ECV^99^ that separated the gWT and resistant populations making them appropriate for use. An interesting observation in this study was the relatively high proportion of strains with moxifloxacin MICs at *gyrA* codon 90 RAVs having MICs of 1–2 µg/mL while those with *gyrA* codon 94 RAVs had MICs of ≥4 µg/mL. The use of high dose moxifloxacin has been suggested to potentially treat strains with an MIC of 1–2 µg/mL in some patients^21^ and does offer hope to retain an important core second line drug especially when treatment options are limited. A second interesting observation was the differential susceptibility pattern among *gyrB* RAVs, though numbers were small and more data would be required to confirm this pattern. This does however raise the need to consider individual drug testing in selected patient groups and the added value of a 96 well plate BMD method, where, simultaneous multi-drug testing is simpler compared with agar or MGIT 960, thus facilitating universal quantitative DST.

Linezolid resistance is uncommon and only three genotypically resistant strains were observed in this study. The criteria established accurately separated susceptible and resistant strains. This drug has not been widely used, as it is expensive and does have a high adverse event profile^22^. This possibly explains the limited number of resistance isolates we found. Furthermore, acquired drug resistance to Linezolid has been shown to be prevented when using current recommended doses of 300 mg or 600 mg correlating with a susceptibility cut off value of 2 ug/mL^23^. As this drug has moved to category “A” forming the backbone of drug resistant TB regimens, greater vigilance for emergent resistance testing is needed, and the BMD is an appropriate method providing MIC data. This method would also be advantageous as the separation between genotypic resistant and gWT was distinct and monitoring gradual increases in MIC overtime would be important.

Clofazimine is a repurposed drug used for treatment of DR-TB and is now included as a category B drug by WHO to always be included where possible. The ECV^99^ ascertained was 0.25 µg/mL, which is consistent with what we previously published^17^, correctly classifying 8 of 9 RAVs in this study. The *Rv0678* gene encodes for an efflux pump and thus variances in MIC are expected dependent on drug exposure. We have previously shown this to be the case for bedaquiline^17^ and may also explain the occurrence of the mutant with a clofazimine MIC in the WT range in this study. Although not described here, bedaquiline testing can be performed on the same plate and performs well, achieving universal DST with a single assay.

There have been several previous studies evaluating the standard format MYCOTB plate, but often these have been applied as comparisons with other methods while applying the criteria of the comparator for the BMD^9–11^. The resultant discordances particularly with strains close to the cut-off used, led Lee and colleagues to propose allowing a 1-dilution variance between methods^24^. This would be overcome if method specific criterion were applied following standardised approaches such as that provided by regulatory authorities and as used in this study. Another area of future research is applying the MICs derived from this assay in clinical cohorts with PK/PD sampling. It is encouraging to note that a clinical trial is planned^25^ aiming to address this gap, however, it is a single country study and multi-country data are still required. Additionally, triangulating the PK/PD findings with the ECV based methods to ascertain clinical breakpoints is what is ultimately required.

Our findings have provided a first step in standardised reporting criteria for the BMD method but needs to be seen in context of the specific limitations. This is a single country study and may not fully represent strains in other parts of the world. However, from reviews of MIC data using other methods conducted by WHO, the distributions have been similar^26^. Nonetheless, multi-country studies are required and these are currently underway. The strains that appeared to be outliers were not re-tested and although it would have been preferable, it does provide a more realistic routine scenario. In conclusion, the current study despite the noted limitations has provided robust validated criteria that will facilitate wider use of BMD as a method for quantitative pDST for Mtb. This will hopefully facilitate progress towards individualised precision medicine for TB and DR-TB. The value of quantitative pDST has also been demonstrated in this study to more accurately interpret mutation data and predict the potential range of MICs.

## Methods

**Figure 4:**
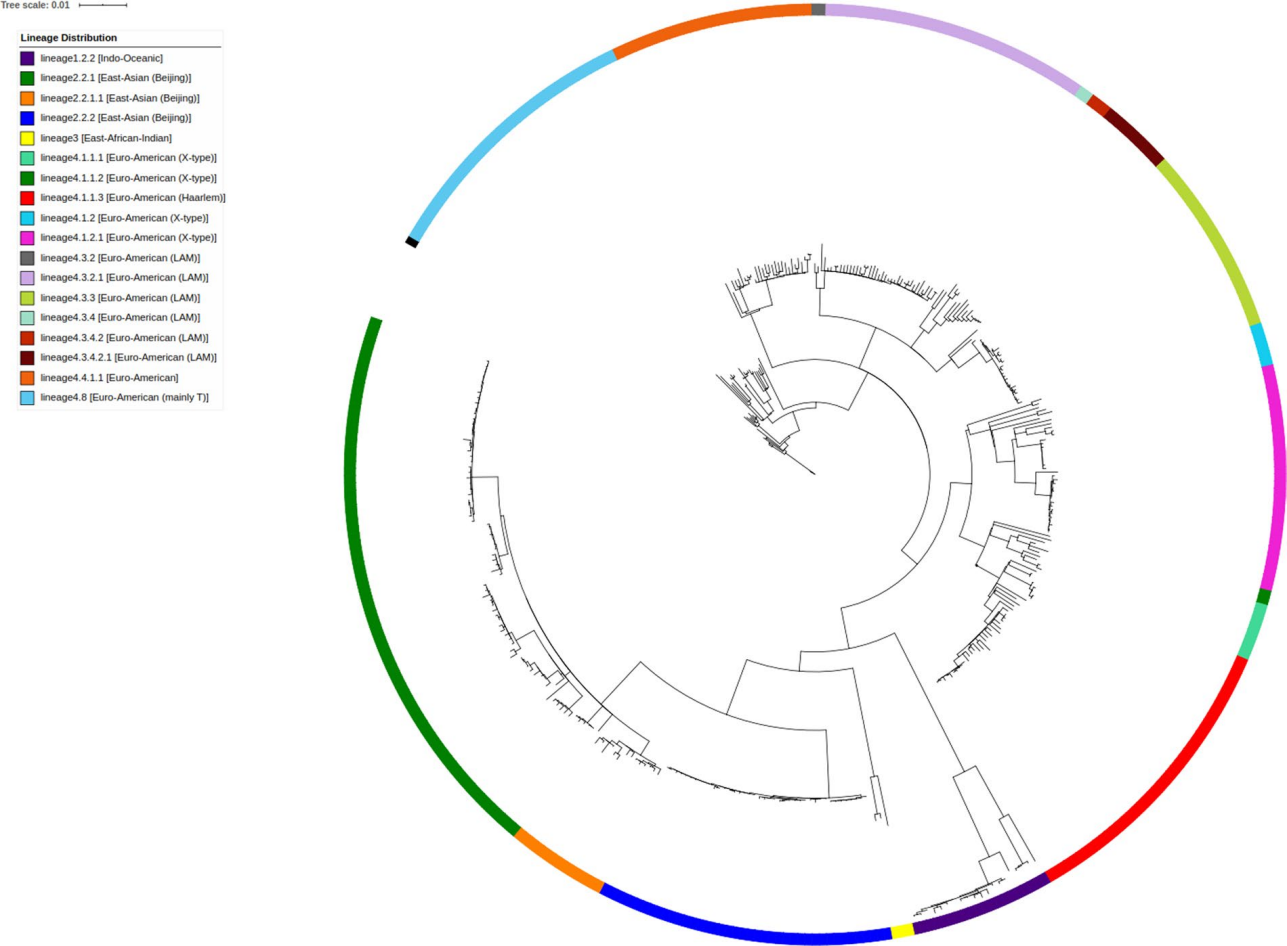
Phylogenetic clustering of strains included in the study and associated lineages (N = 385).

*Mycobacterium tuberculosis* strains which are broadly representative of strains circulating in South Africa were used. These strains were previously used for the determination of bedaquiline interpretive criteria on multiple methods including the commercial BMD plate^17^. In this study, we analysed the results of the other available anti-mycobacterial drugs on the plate which included: rifampicin (rif), isoniazid (inh), ethambutol (emb), ofloxacin (ofx), moxifloxacin (mxf), levofloxacin (levo), amikacin (ami), kanamycin (kana), capreomycin (cap), clofazimine (cfz) and linezolid (lzd), all key drugs for both first and second line treatment of Mtb. Of the 391 strains selected for testing, six had >3 drug inconsistencies between phenotypic and genotypic results and were excluded due to possible technical errors. Of the 385 strains with valid results, 68 (18%) were rifampicin susceptible, 317 (82%) were rifampicin resistant (RR) or MDR. Of the RR/MDR strains, 109 (34%) were pre-XDR/XDR, being MDR-TB with either fluoroquinolone or second line injectable resistance, or both, respectively. The strains represented the common lineages and were diverse (Fig. 4). The BMD plates were prepared and shipped frozen from the manufacturer in accordance with Food and Drug Agency (FDA) requirements.

The isolates were tested on BMD and had whole genome sequencing (WGS) performed as previously described^17,27^. In brief, WGS was performed using the MiSeq (Illumina, UK). Library preparation was performed using the Nextera-XT library preparation kit (Illumina, UK) and sequencing performed using the 2 × 300 bp MiSeq cartridge v.3 (Illumina, UK) with a target of 30×–50× paired coverage (~80–100X coverage). CLC Genomics Workbench 8.5.1(Qiagen, Venlo, The Netherlands) was used to detect RAVs using Reference mapping against the annotated reference genome H37Rv (NC00962.3) and the quality-based variant analysis tools where SNPs were filtered and qualified. Association of mutations as resistance predictors were primarily identified using the TB Drug Resistance Mutation Database (TBDReaMDB)^28^. If a mutation was not listed, literature, including newer published databases such as TBProfiler and PhyResSE, was surveyed to identify the association^29,30^. *Mycobacterium tuberculosis* lineages were assigned using the Single Nucleotide Polymorphism (SNP) barcode described by Coll *et al*.^31^. Individual SNP files for isolates were generated using CLC Genomics workbench v 8.5.1 (Qiagen, Venlo, The Nederlands). The SNPs for each genome were concatenated to an alignment and phylogeny inferred based on a comparison of SNP alignments of strains. SNP alignments were analysed using IQ-TREE (default settings) to generate a maximum-likelihood phylogenetic tree^32^. The output was visualized and lineage annotated with ITOL v.4.4.1^33^.

The epidemiological cut off values (ECV) for all drugs were determined using the ECOFF finder^34^. For the ECV determination, we used strains that were genotypically wild type (gWT) considering well-known resistance associated variants (RAVs). A-priori an ECV of 99% (ECV^99^) was selected which is recommended by regulatory bodies (EUCAST and CLSI) and adopted by WHO recently. The ECV^99^ is the MIC value identifying the upper limit (99%) of the wild type population. The derived ECVs were evaluated for each drug and compared to the WGS data for the presence of mutations with known resistance associated variants (RAVs) and related MICs to validate the appropriateness of the ECV^99^ selected. The genetic targets for the following drug and gene combinations were analysed: rifampicin (*rpoB)*; isoniazid (*katG, inhA, ahpC, ahpC promoter* and *fabG1 promoter)*; ethambutol (*embB)*; levofloxacin/moxifloxacin (*gyrA* and *gyrB)*; amikacin/kanamycin/capreomycin (*rrs)*, kanamycin (*eis)*, linezolid (*rplC* and *rrL)*, clofazimine (*Rv0678)*. For clarity of interpretation, RAVs for a specific drug were analysed against strains with only a single gene having RAVs, e.g. strains with *gyrA* RAVs were analysed having no *gyrB* RAVs. However, for isoniazid, more than one RAV is frequent and we present distributions for both single and dual RAVs.

## Supplementary information

Supplementary Information.

## Data Availability

The data presented in this manuscript are available in the European Nucleotide Archive under accession number: PRJEB25997.
