# Supplementary material for: Epidemiological cut-offs for Sensititre susceptibility testing of Mycobacterium tuberculosis: interpretive criteria cross validated with whole genome sequencing
Source: Sci Rep. 2020 Jan 23;10:1013. doi: 10.1038/s41598-020-57992-x (PMC6978314; doi:10.1038/s41598-020-57992-x)
Supplement: Supplementary file 1 — Supplementary Information. [file 41598_2020_57992_MOESM1_ESM.docx]

**Supplementary Information**

**Title:** Epidemiological cut-offs for Sensititre susceptibility testing of *Mycobacterium tuberculosis*: interpretive criteria cross validated with whole genome sequencing

**Authors:**

*Nazir A Ismail^a,b,c^, Farzana Ismail^a,b^, Lavania Joseph^a^, Netricia Govender^a^, Linsay Blows^a^, , Koné Kaniga^d^, Shaheed V Omar^a^

^a^National Institute for Communicable Diseases, Centre for Tuberculosis, Johannesburg, South Africa

^b^Department of Medical Microbiology, University of Pretoria, Pretoria, South Africa

^c^Department of Internal Medicine, University of Witwatersrand, Johannesburg, South Africa

^d^Janssen Research & Development, Titusville, NJ, United States of America

Corresponding author:

Prof Nazir Ahmed Ismail

Centre for Tuberculosis, National Institute for Communicable Diseases,

A division of the National Health Laboratory Service

1 Modderfontein Road, Sandringham, Johannesburg 2131, South Africa

Office: +27-11-8855-321

Cell: +27-82-6000-857

Email: [naziri@nicd.ac.za](mailto:naziri@nicd.ac.za)

Table S1: Proportion of false susceptible results due to presence of RAVs applying the ECV^99^

| Drug | ECV^99^ (µg/mL) | No. isolates with a RAV | No. false susceptible | % false susceptible |
| --- | --- | --- | --- | --- |
| Rifampicin | 0.25 | 321 | 4 | 1% |
| Isoniazid | 0.25 | 252 | 17 | 7% |
| Ethambutol | 4 | 207 | 1 | 0% |
| Levofloxacin | 1 | 107 | 4 | 3% |
| Moxifloxacin | 1 | 107 | 3 | 3% |
| Amikacin | 4 | 53 | 0 | 0% |
| Kanamycin | 8 | 59 | 0 | 0% |
| Capreomycin | 8 | 53 | 0 | 0% |
| Linezolid | 4 | 3 | 0 | 0% |
| Clofazimine | 0.25 | 9 | 1 | 11% |

ECV^99:^ Epidemiological cut-off value (99%)

RAV: Resistance associated variant

Table S2: Distribution of MICs and RAVs for selected drugs with mutation data

* Negative (-) refers to upstream region from gene start. Green shading - drug susceptible MIC range. Blue shading - drug susceptible MIC range overlapping with RAVs for isoniazid

Table S3: Distribution of MICs and RAVs for moxifloxacin and levofloxacin with mutation data

MIC: Minimum inhibitory concentration. RAV: Resistance associated variant

Figure S1: Wild-type ECV estimation using iterative non-linear regression on expanding subsets for rifampicin (N=65)

Figure S2: Wild-type ECV estimation using iterative non-linear regression on expanding subsets for isoniazid (N=134)

Figure S3: Wild-type ECV estimation using iterative non-linear regression on expanding subsets for ethambutol (N=179)

Figure S4: Wild-type ECV estimation using iterative non-linear regression on expanding subsets for moxifloxacin (N=278)

Figure S5: Wild-type ECV estimation using iterative non-linear regression on expanding subsets for levofloxacin (N=278)

Figure S6: Wild-type ECV estimation using iterative non-linear regression on expanding subsets for amikacin (N=263)

Figure S7: Wild-type ECV estimation using iterative non-linear regression on expanding subsets for kanamycin (N=263)

Figure S8: Wild-type ECV estimation using iterative non-linear regression on expanding subsets for capreomycin (N=263)

Figure S9: Wild-type ECV estimation using iterative non-linear regression on expanding subsets for clofazimine (N=272)

Figure S10: Wild-type ECV estimation using iterative non-linear regression on expanding subsets for linezolid (N=382)
